# Correction to “Assessment of Patient Safety Culture Among Healthcare Providers in Tertiary Hospitals in Malaysia—A Cross‐Sectional Study”

**DOI:** 10.1002/hsr2.70229

**Published:** 2024-12-02

C. Sok May, P. Sivanandy, P. V. Ingle, and P. Manirajan, “Assessment of Patient Safety Culture Among Healthcare Providers in Tertiary Hospitals in Malaysia—A Cross‐Sectional Study,” *Health Science Report* 7 (2024): e70035, https://doi.org/10.1002/hsr2.70035.

In “Assessment of patient safety culture among healthcare providers in tertiary hospitals in Malaysia—A cross‐sectional study,” which was published in volume 7, issue 10, October 2024, the names of the hospitals where the studies were conducted have been removed to preserve anonymity. This change does not affect the overall conclusions.

